# Metastability, an emerging concept governing BOK-mediated apoptosis initiation

**DOI:** 10.18632/oncotarget.25801

**Published:** 2018-07-24

**Authors:** Tudor Moldoveanu, Janet H. Zheng

**Affiliations:** Tudor Moldoveanu: Departments of Structural Biology & Chemical Biology and Therapeutics, St. Jude Children’s Research Hospital, Memphis, TN, USA

**Keywords:** apoptosis, membrane permeabilization, BCL-2 proteins, BOK, metastability

By regulating mitochondrial outer membrane permeabilization (MOMP), the rate-limiting event in caspase activation, BCL-2 family proteins are responsible for apoptosis initiation [1]. Most BCL-2 proteins obey canonical rules of mitochondrial apoptosis [2]. These rules define the intricate protein–protein interactions among the different pro- and anti-apoptotic proteins to dictate cell fate [3]. The BCL-2-related ovarian killer (BOK) is unusual as it does not play by these rules. For instance, unlike the canonical effectors BAK and BAX, BOK does not require direct activation by the BCL-2 homology 3 (BH3)-only proteins, and is not inhibited by anti-apoptotic BCL-2 proteins [4]. Instead, BOK is constantly shredded by the gp78 E3 ligase–proteasome system to which maintain an undetectable its cellular level [4]. Accordingly, BOK’s role in apoptosis has been revealed upon inhibition of BOK degradation or under ER stress [4]. Although BOK has been definitively implicated in mitochondrial apoptosis initiation in the absence of BAK and BAX as a bona fide effector of MOMP [4, 5], another study casted some doubt over BOK’s independence of regulation by BH3-only proteins regulation of MOMP and its ability to permeabilize mitochondria [6]. Nonetheless, that study corroborated BOK’s ability to permeabilize liposomes and suggested a preference of BOK for the negatively charged phospholipid cardiolipin for efficient membrane permeabilization [6]. Given these unresolved matters, we sought to mechanistically probe BOK-mediated initiation of apoptosis based on a comprehensive structure-function analysis [7].

**Figure 1:**
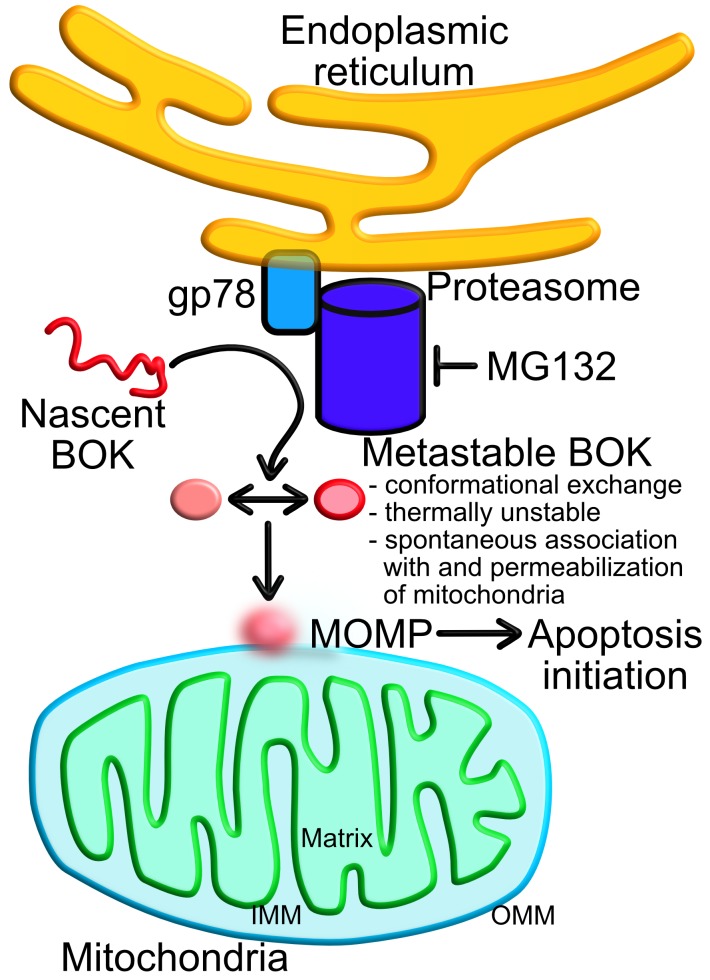
BOK is a metastable effector of MOMP Nascent BOK is constantly degraded by the endoplasmic reticulum associated degradation E3 ligase gp78–proteasome system. Proteasome inhibition with MG132 results in BOK accumulation. BOK folds similar to other globular BCL-2 family proteins, but it adopts multiple conformations (depicted as two states captured at high-resolution) in its dynamic regulatory hydrophobic groove. In addition to the shape-shifting groove, which undergoes conformational exchange detectable by NMR, BOK has a destabilized regulatory helix α1. These features contribute to its metastability or the ability to adopt different conformations. Thermal instability and spontaneous association with and permeabilization of mitochondria support BOK’s metastability, which explains its independence of BH3-only activators. MOMP conformations are unresolved (blurred). IMM, inner mitochondrial membrane; OMM, outer mitochondrial membrane.

First, we engineered human BOK protein and determined its structure by nuclear magnetic resonance (NMR) to reveal a typical BCL-2 fold with a ligand-binding groove made up of mostly loop structures that anchor the one turn helix α3 to α2 and α4. This is a flexible region exhibiting dynamics detectable by NMR suggesting that it undergoes conformational exchange, or shape shifting (Figure 1). Corroborating our observations, in the recently determined crystal structure of the BCL-2 core of chicken BOK, two BOK monomers observed in the crystal asymmetric unit exhibited distinct conformations in the hydrophobic groove – one resembling that of the NMR structure and another with α3 wound into a 2-turn helix (Figure 1) [8]. With these studies, we now have access to the entire repertoire BCL-2 family folded protein structures.

NMR-based binding analysis of BID BH3 to BOK suggested weak affinity (K_D_ 30-300 µM) that was pH and temperature dependent. Specific BID BH3 binding to the hydrophobic groove of BOK was similar to that observed in the BID stapled alpha helix of BCL-2 proteins (SAHB)–BAK complex [9]. While we detected activation of BOK by BID BH3 under certain dosing combinations in liposome permeabilization, the same effect was not observed in purified mitochondria, which was otherwise readily permeabilized by recombinant BOK at low µM concentrations. We demonstrated that BOK is independent of BH3 ligands in cells. tBID, the activated form of BID and one of the most potent direct activators of BAK and BAX, was unable to promote any additional apoptosis in BOK-expressing *bak*^*-/-*^ *bax*^*-/-*^ mouse embryonic fibroblasts beyond level induced by BOK stabilization with the proteasome inhibitor MG132 (Figure 1). We currently do not know if other BH3-only proteins would be able to accelerate BOK-mediated cell death upon proteasome inhibition.

How is BOK activated? We propose that BOK is predisposed to spontaneous unfolding, which manifests in the presence of target membranes. This is consistent with BOK thermal instability, which correlates with spontaneous mitochondrial association and permeabilization (Figure 1). In contrast, BAK is significantly more thermally stable, and does not exhibit spontaneous mitochondrial association and permeabilization. We identified two structural elements that predispose BOK to spontaneous unfolding. 1) The shape-shifting hydrophobic groove, and 2) an intrinsically unstable helix α1, which bears a destabilizing glycine in the middle of the helix. One of the monomers observed in the chicken BOK crystal structure exhibited a hole through the core of the protein that has also been observed in complexes between BAK and BAX with BH3 peptides [8]. Ligand-induced holes have been assumed to be destabilizing, and BOK may exhibit this structural feature even without ligand binding. Alanine substitution of the destabilizing glycine in α1, significantly increased the thermal and chemical stability of BOK, and substantially inhibited liposome permeabilization, MOMP, and apoptosis compared to WT BOK. Based on these unique features, we coined the term metastability, or the ability to easily adopt multiple states, to define a new paradigm for BOK-mediated MOMP (Figure 1), which may also apply to BAK and BAX upon direct activation.

Our work reveals intrinsic structural features that facilitate BOK auto-activation. But, how do BOK, BAK, and BAX permeabilize membranes remains unsatisfactorily defined (Figure 1) [10]. Additionally, while the role of BOK *in vivo* as effector of MOMP is now unequivocal [8], we have not yet identified triggers beyond targeting its protein turnover. Has BOK evolved as a lone wolf of apoptosis to bestow a fail-safe mechanism for endoplasmic reticulum associated degradation (ERAD) machinery? This machinery is crucial to cells, tissues, organs and organisms, none of which can afford to lose it or they face dysregulation and disease. Future research in this rich and essential area will address these problems.
